# The Canine Papillomavirus and Gamma HPV E7 Proteins Use an Alternative Domain to Bind and Destabilize the Retinoblastoma Protein

**DOI:** 10.1371/journal.ppat.1001089

**Published:** 2010-09-02

**Authors:** Jingang Wang, Dan Zhou, Anjali Prabhu, Richard Schlegel, Hang Yuan

**Affiliations:** Department of Pathology, Georgetown University Medical School, Washington, D.C., United States of America; Brigham and Women's Hospital/Harvard Medical School, United States of America

## Abstract

The high-risk HPV E6 and E7 proteins cooperate to immortalize primary human cervical cells and the E7 protein can independently transform fibroblasts in vitro, primarily due to its ability to associate with and degrade the retinoblastoma tumor suppressor protein, pRb. The binding of E7 to pRb is mediated by a conserved Leu-X-Cys-X-Glu (LXCXE) motif in the conserved region 2 (CR2) of E7 and this domain is both necessary and sufficient for E7/pRb association. In the current study, we report that the E7 protein of the malignancy-associated canine papillomavirus type 2 encodes an E7 protein that has serine substituted for cysteine in the LXCXE motif. In HPV, this substitution in E7 abrogates pRb binding and degradation. However, despite variation at this critical site, the canine papillomavirus E7 protein still bound and degraded pRb. Even complete deletion of the LXSXE domain of canine E7 failed to interfere with binding to pRb in vitro and in vivo. Rather, the dominant binding site for pRb mapped to the C-terminal domain of canine E7. Finally, while the CR1 and CR2 domains of HPV E7 are sufficient for degradation of pRb, the C-terminal region of canine E7 was also required for pRb degradation. Screening of HPV genome sequences revealed that the LXSXE motif of the canine E7 protein was also present in the gamma HPVs and we demonstrate that the gamma HPV-4 E7 protein also binds pRb in a similar way. It appears, therefore, that the type 2 canine PV and gamma-type HPVs not only share similar properties with respect to tissue specificity and association with immunosuppression, but also the mechanism by which their E7 proteins interact with pRb.

## Author Summary

Human papillomaviruses (HPVs) are estimated to cause the most common sexually transmitted infection in the world, and these infections are recognized as the major cause of cervical cancer. One of the papillomavirus oncoproteins, E7, plays a major role in both the viral life cycle and progression to cancer. In cells E7 associates and inactivates pRb, a tumor suppressor protein. For the vast majority of papillomaviruses, E7 binds to pRb using a small amino acid sequence, LXCXE. However, we have now identified a papillomavirus E7 protein that lacks the LXCXE domain yet still binds and degrades pRb. This E7 protein, derived from a carcinogenic canine virus, uses its C-terminal domain to bind pRb. In addition, we discovered that a family of papillomaviruses, the gamma type HPVs, also lacks the LXCXE domain and binds pRb using a similar mechanism.

## Introduction

Human papillomaviruses (HPVs) mediate the initiation and maintenance of cervical cancer [1], [2]. Based upon DNA sequence homology, there are more than 150 different HPV genotypes, 40 of which infect anogenital and oral mucosa [3]. In addition to genotyping, HPVs can also be classified as low-risk and high-risk based on the clinical prognosis of their associated lesions. Low-risk HPVs cause benign epithelial hyperplasias while high-risk HPVs cause lesions that can progress to malignancy. Integration of the HPV genome into a host cell chromosome is a frequent event during malignant progression and it may play a significant role in dysregulated expression of the HPV E6 and E7 proteins [4]. The high-risk HPV E6 binds to several cell targets, including p53, Myc, E6AP, PDZ proteins and other cellular proteins to alter apoptotic/growth regulatory pathways and induce cellular telomerase activity [5]. The E7 protein binds and sequesters pRb and directs its ubiquitin-mediated proteolysis [6], thereby altering E2F-regulated genes and allowing cells to enter the S phase of the cell cycle.

The E7 oncoprotein is approximately 100 amino acids in length and contains two highly conserved regions (CRs), the amino-terminal CR1 and CR2 domains [7]. The E7 CR1 and CR2 domains share strikingly high homology with the CR1 and CR2 regions of adenovirus (Ad) E1A and related sequences in simian vacuolating virus 40 (SV40) large tumor antigen (T Ag) [4], [8]. For each of these viruses, the CRs contribute significantly to cell transformation [9], [10], [11], [12]. A conserved Leu-X-Cys-X-Glu (LXCXE) motif in the CR2 domain of HPV E7, as well the ones in Adenovirus E1A and SV40 LT, are necessary and sufficient for association with pRb [13]. The crystal structure of pRb bound to an E7 peptide was resolved, and revealed that LXCXE of HPV E7 binds entirely through the B domain of pRb [14]. For high risk HPV, the LXCXE motif is also required for pRb degradation[15], [16]. The carboxyl-terminal domain of E7 consists of a metal binding domain composed of two CXXC motifs separated from each other by 29 amino acids [14]. This zinc-binding region is important for E7 dimerization and intracellular stabilization [10], [17]. The carboxyl-terminal domain also contributes to E7 association with chromatin-modifying enzymes, particularly histone deacetylases and histone acetyl transferases [18]. Although the carboxyl-terminus of high-risk HPV E7 does not appear to have a direct role in the binding and degradation of pRb [15], [19], it has been proposed to be important for releasing E2F from pRb [20], [21].

Papillomavirus can be isolated from a wide range of vertebrates, ranging from birds to manatees [22], [23] and infection by these viruses is, in general, species-specific. The canine papillomavirus model has been used successfully for vaccine [24], [25] and therapeutics studies [26]. Recently, our lab isolated and sequenced canine papillomavirus type 2 (CPV-2, previously named CfPV2), which we showed to be an epidermotropic virus that occurred frequently in immunosuppressed animals and induced tumors that progressed to aggressive cancers [27], [28]. The E7 gene of CPV-2 appeared unique in that it lacked the conserved LXCXE motif. In this study we show that this variant E7 protein is still able to bind and degrade pRb and that the primary domain for binding pRb is in its carboxyl-terminus. Interestingly, upon searching the HPV genome database, we observed that the gamma HPVs also contained the variant LXSXE E7 domain and, similar to the canine E7 protein, could still bind and degrade pRb. In addition to this similarity, we also noted that both the type 2 canine PV and gamma HPVs both exhibited a tropism for skin and for immunocompromised hosts.

## Materials and Methods

### Plasmids

Wild-type CPV-2 E6 and E7 were generated by PCR using CPV-2 genome as template [27] and subcloned into retrovirus vector pLXSN (Clontech) at the sites *EcoR I and BamH I*. The CPV-2 E7 mutants CPV-2 E7 ΔLXSXE, S26C, and S26G were generated by using the QuikChange Site-Directed Mutagenesis kit (Stratagene), and CR1, CR2, CR1CR2 and CT were generated by PCR using pLXSN.CPV-2E7 as template. All the wild type E7 and mutants were cloned into the sites *EcoR I and Not I* of the pGEX4T-2 (GE Healthcare) for GST fusion protein expression. CPV-2 E7 and mutants with a hemagglutinin (HA) epitope tag at their amino terminal or carboxyl terminal were generated by PCR. PCR products were then subcloned into the mammalian expression vector pJS55 [29] at the sites *EcoR I and BamH I*. All plasmids were sequenced to confirm the presence of corresponding mutations. All primer sequences used in subcloning and site-directed mutagenesis please see Supporting Information S1. All the wild type and mutants of HPV-4 E7 DNA (GenBank NC_001457.1) were synthesized (Celtek Bioscience), and cloned into pGEX4T-2 (GE Healthcare) for GST fusion protein expression.

### Cell lines and culture

U2OS cells, Hela cells and SD3443 cells were maintained in Dulbecco's Modified Eagle's Medium (DMEM) (Invitrogen) supplemented with 10% Fetal Bovine Serum (FBS). Primary human keratinocytes were derived from neonatal foreskins as described previously [30] and were grown in Keratinocyte-SFM medium (Invitrogen).

### Transfection and retrovirus Infection

U2OS cells were co-transfected with RcCMV-Rb and pJs55, pJS55-HA.HPV16 E7, pJS55-HA.CPV-2 E7ΔLXSXE, pJS55-HA.CPV-2 E7CR1CR2, pJS55-HA.CPV-2 E7C-RT or pJS55-HA.CPV-2 E7 using Lipofectamine 2000 (Invitrogen) as specified by the manufacturer. Hela cells were transfected with pJs55, pJS55-HA.HPV16 E7 or pJS55-HA.CPV-2 E7 using Lipofectamine 2000 (Invitrogen) as specified by the manufacturer. Cell were harvested and lysed by RIPA buffer (25 mM Tris•HCl pH 7.6, 150 mM NaCl, 1% NP-40, 1% sodium deoxycholate, 0.1% SDS) 24 hours post transfection.

To prepare retrovirus stocks, SD3443 cells were transfected with E7 retrovirus constructs using Fugen (Roche applied science, US.) as specified by the manufacturer. Culture supernatants containing retrovirus were collected 48 h post-transfection. Viral titers of the supernatants were determined using 3T3 cells. The primary HFK cells (passage 0) were infected at a multiplicity of 10 PFU/cell with retrovirus expressing wild type E6, E7 or E7 mutants. Retrovirus-infected cells were selected in G418 (50 ng/ml) for 2 days.

### GST fusion protein expression and purification

GST and GSTE7 fusion proteins were expressed in BL21pLysS cells (Invitrogen). The cells were induced with 100 µM isopropyl-β-D-thiogalactopyranoside (IPTG) 6 hours at 25°C once the optical density at 600 nm reached 0.8–1.0. Recombinant CPV-2 E7 and mutants were purified from the supernatant of disrupted cells by glutathione-Sepharose chromatography as previously described [24].

### Preparation of cell extracts and Western blot analysis

Proteins were extracted from cells and measured concentration as previously described[31]. Proteins were separated on a 4 to 20% Tris-glycine gradient gel (Novex) and then were electrophoretically transferred to an Immobilon-P polyvinylidane difluorid (PVDF) membrane (Millipore). The primary antibody was used at a dilution of 1∶1,000 or 1∶3,000. The secondary antibodies, alkaline phosphatase-conjugated goat anti-mouse IgG and anti-rabbit IgG (Tropix) antibodies, were used at a dilution of 1∶2,000. Western blots were visualized by using SuperSignal West Pico Chemiluminescent Substrate (Thermo Scientific). The following commercial antibodies were used: for pRb (1∶1000 dilution), Rb (4H1) Mouse mAb (Cell Signaling technology); for glutathione S-transferase (1∶3000 dilution), catalog no. 3818-1 (Clontech); for HA (1∶1000 dilution), HA.11 clone 16B12 (Covance).

### Analysis of protein interactions

For GST pull-down assays, Jurkat cell or CPEK cell nuclear extract (50 µg) was incubated with 5 µg of GST or GST fusion protein in binding buffer [20 mM Hepes/150 mM KCl/4 mM MgCl2/1 mM EDTA/0.02% Nonidet P-40/10% glycerol/0.035% 2-mercaptoethanol/1% (vol/vol) Sigma protease inhibitor mixture] and rocked for 1 h at 4°C. Glutathione-Sepharose beads (Amersham Pharmacia Biosciences) were added to each reaction and rocked for another 1 h at 4°C. The beads were then washed with 1 ml of washing buffer (125 mM Tris, 150 mM NaCl, pH 8.0) four times and boiled with 2× SDS sample buffer, and the proteins were separated by SDS/PAGE. Western blots were used to measure the level of pRb and GSTE7 proteins. The bands of pRb and GSTE7 proteins were quantified by densitometry using Quantity One (BioRad). The relative binding activities were calculated using pRb bound by wild type CPV-2 E7 GST fusion protein as 100%, and normalized with GSTE7 bands.

For co-immunoprecipitation assays, N-terminal HA-tagged CPV-2 E7 proteins were immunoprecipitated with a polyclonal anti-pRb antibody (Santa cruz). C-terminal HA-tagged CPV-2 or HPV16 E7 proteins were immunoprecipitated with a polyclonal anti-HA antibody (Santa Cruz). Bead washing buffer was 25 mM Tris•HCl pH 7.6, 150 mM NaCl, 1% NP-40, 1% sodium deoxycholate, 0.1% SDS, 1% Sigma protease inhibitor mixture. The pulled down complexes were resolved on a 4 to 20% gradient gel and then analyzed by Western blotting using either anti-pRb antibody, anti-HA antibody or anti-cullin2 antibody (Invitrogen).

### Reverse transcription PCR

Cells in a 3.5 cm diameter dish were lysed with 1 ml TRIZOL (Invitrogen). Total RNA was isolated according to manufacturer's protocol. Reverse transcription PCR was performed using ONE STEP RT-PCR KIT (QIAGEN) as specified by the manufacturer. All primer sequences and condition please see Supporting Information S1.

### Protein sequence analysis

Multiple sequence alignments of E7 were prepared using Clustal W. The phyllogenetic analysis was conducted using the Mega version 4.0 [32].

## Results

### Despite lacking the conserved LXCXE motif, CPV-2 E7 can still degrade pRb in cells

**Figure 1:**
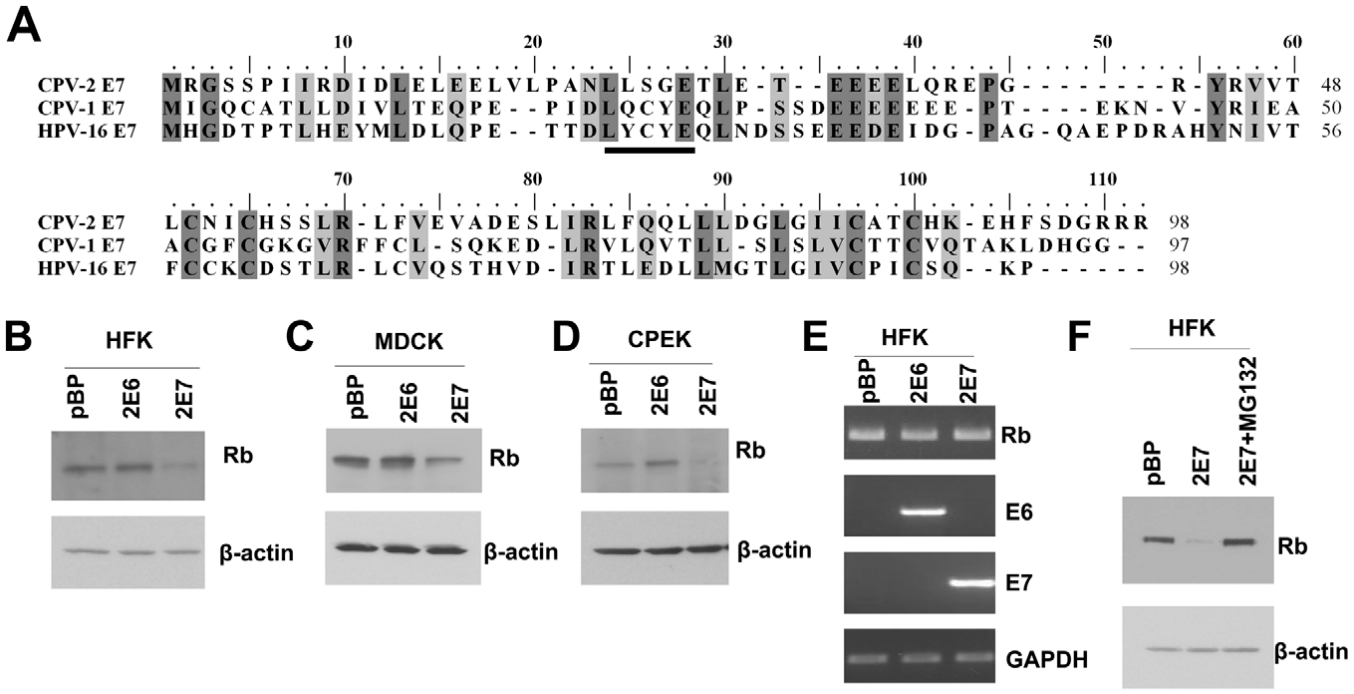
Despite the lack of a conserved LXCXE motif, CPV-2 E7 still degrades pRb. (A) Alignment of CPV-2 E7, CPV-1E7 and HPV16 E7 protein sequences. The underlined sequence is the conserved LXCXE domain. (B-D) Human foreskin keratinocytes (HFKs), canine MDCK cells or canine keratinocytes (CPEKs) were infected with control retrovirus or retrovirus encoding for CPV-2 E6 (2E6) or E7 (2E7). Cell lysates were prepared from indicated cells. Proteins were separated on a 4 to 20% gradient gel and then analyzed by Western blotting using anti-pRb antibody. (E) RNA was isolated from indicated cells, and RT-PCR was used to measure the level of RNA transcripts. (F) Cell lysates were prepared from cells treated with control DMSO or 40 um of MG132 for 4 hours. The level of pRb was measured by western blot. In this figure and all following figures, the described experiments were repeated at least three times, with representative results shown.

Several studies have demonstrated that the conserved LXCXE motif in the HPV E7 CR2 domain is necessary and sufficient for binding pRb [4]). Studies have also revealed that the substitution of C or E or complete deletion mutation of the LXCXE motif destroys pRb binding. Without binding to the pRb, E7 is unable to degrade pRb [4]. Our laboratory recently isolated CPV-2 from footpad and interdigital papillomas of immunosuppressed dogs. Sequencing revealed that the CPV-2 E7 protein contained the typical two C-X-X-C motifs within its carboxyl terminal half but lacked the conserved pRb binding site (LXCXE) which is present in COPV (CPV-1) E7 and most HPVs (Figure 1A). CPV-2 E7 has a serine (amino acid 26) in the position of cysteine in the LXCXE motif. In order to test whether CPV-2 E7 could degrade pRb, canine E7 was transduced into human keratinocytes (HFKs), canine kidney cells (MDCKs) or canine keratinocytes (CPEKs) by using retrovirus infection. Cell lysates were collected, and pRb levels were measured by western blots. Surprisingly, despite lacking the conserved LXCXE motif, CPV-2 E7 was still able to degrade pRb in HFKs, MDCKs and CPEKs (Figure 1B, C and D). To test whether the lower level of pRb was due to a change at the transcriptional level, RT-PCR was performed to measure the level of pRb mRNA. There was no significant difference between the amount of pRb mRNA in control cells and cells with CPV-2 E7 (Figure 1E). In addition, treatment of the E7 expressing cells with the proteasome inhibitor, MG132, restored the level of pRb (Figure 1F). These data suggest that the reduction of pRb by CPV-2 E7 occurs at the protein level rather than mRNA level, and that degradation is most likely responsible.

### The LXSXE motif is not required for binding of pRb by CPV-2 E7

**Figure 2:**
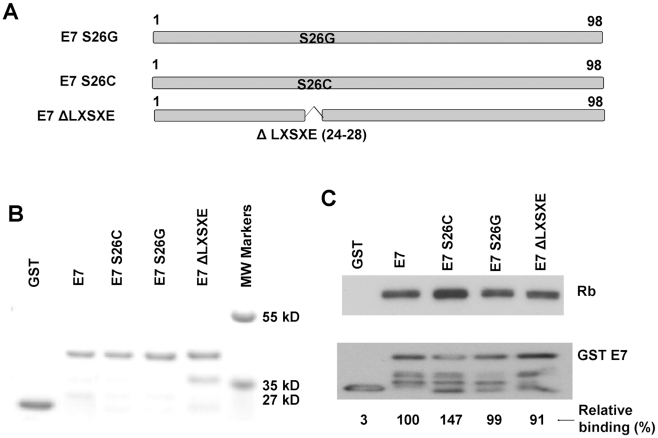
The canine E7 LXSXE motif is not required for pRb binding. (A) Schematic representation of CPV-2 E7 proteins with mutations. (B) Expression of bacterially expressed GST E7 fusion proteins were confirmed by SDS-PAGE and Coomassie blue staining. (C) GST pull-down experiments were used to analyze the binding of GST E7 constructs to pRb. Complexes were resolved on 4–20% gradient gels and then analyzed by Western blotting using anti-pRb antibody or anti-GST antibody. The pRb and GSTE7 proteins were quantified by densitometry. The relative binding activities were normalized to GSTE7 protein and the wild type CPV-2 E7 GST fusion protein was set to 100%.

The degradation of pRb by HPV16 E7 requires high affinity binding [19]. Since CPV-2 E7 lacks the conserved pRb binding motif, LXCXE, there could be two possibilities for the high affinity binding of pRb by CPV-2 E7. It could be either that the LXSXE motif has the same binding properties as LXCXE, or that CPV-2 E7 has an alternative dominant binding site. We generated E7 mutants (Figure 2A) with mutations within the LXSXE domain to investigate whether the LXSXE motif exhibits similar binding to pRb as LXCXE. The GST E7 wild type and mutant fusion proteins were purified from bacteria (Figure 2B), and tested for their binding to pRb. As demonstrated in Figure 2C, wild type CPV-2 E7 binds well to pRb. Substitution of serine26 to cysteine in CPV-2 E7 significantly increased the interaction between E7 and pRb. This is in agreement with mutagenesis studies showing that mutation of HPV16 E7 cysteine24 to serine substantially decreased the binding activity [13], [33]. In the studies of HPV16 E7, the substitution of cysteine in LXCXE for glycine abolished the binding between E7 and pRb [14], [33], [34], [35]. However, for CPV-2 E7, the pRb binding activity of the glycine mutant protein was still retained (Figure 2C). More importantly, the deletion of the entire LXSXE motif did not significantly affect the ability of CPV-2 E7 to bind pRb. This is very surprising since the deletion of the LXCXE motif in HPV 16 E7 totally abolished pRb binding [14], [33], [34], [35]. Thus, while the CPV-2 LXSXE motif exhibits lower pRb binding than the conserved LXCXE motif, it is clear that there is an alternative pRb binding site in the CPV-2 E7 protein.

### In vitro, the CPV-2 E7 carboxyl-terminal domain mediates pRb binding

**Figure 3:**
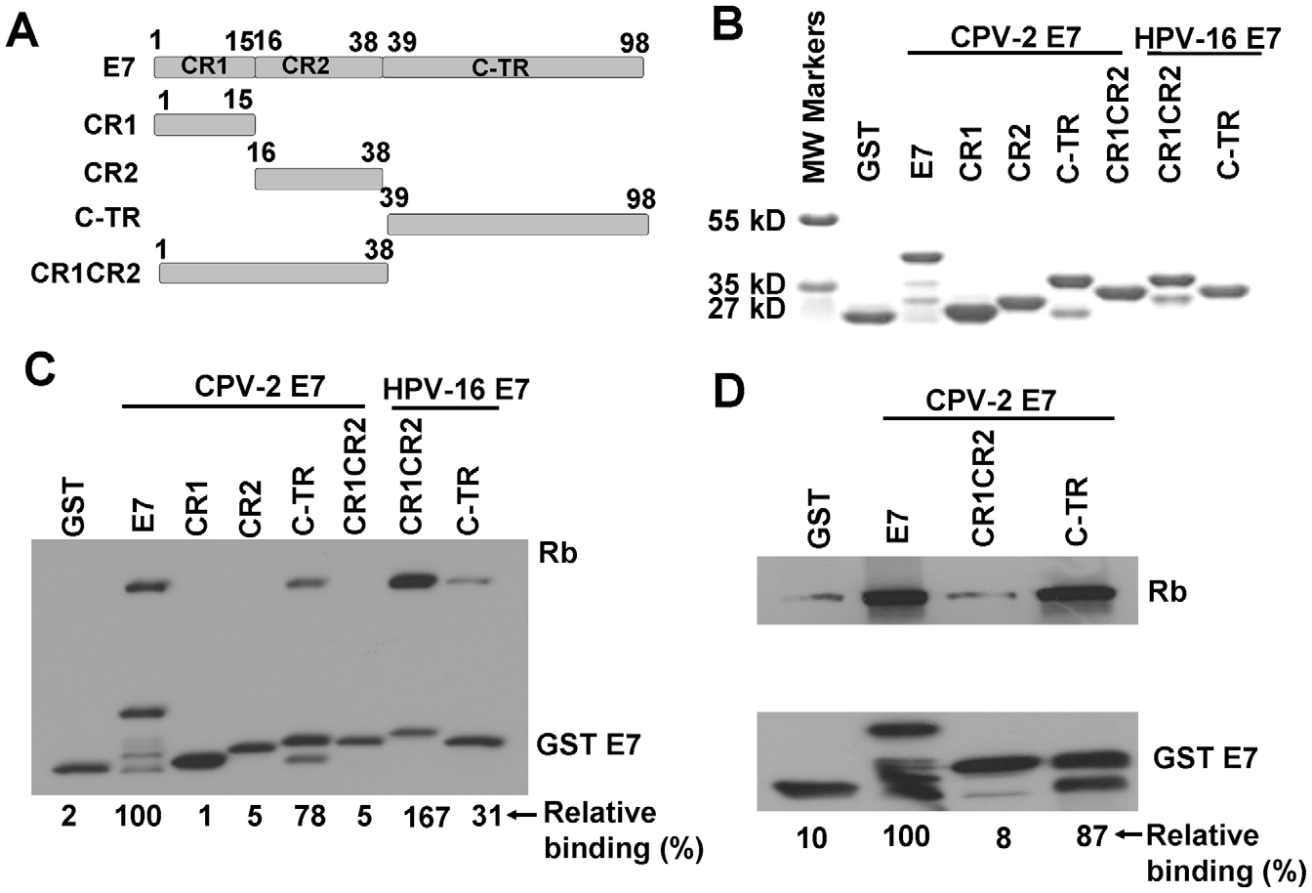
The carboxyl-terminus of canine E7 protein mediates pRb binding in vitro. (A) Schematic representation of CPV-2 E7 proteins with mutations. CPV-2 E7 protein was divided into CR1 (amino acids 1 to 15), CR2 (amino acids 16 to 38) and C terminal domains (amino acids 39 to 98). (B) Expression of bacterially expressed GST tagged E7 truncated mutants were confirmed by SDS-PAGE and Coomassie blue staining. GST pull-down experiments were used to analyze the binding of GST E7 constructs to human pRb (C) and canine pRb (D). Complexes were resolved on 4–20% gradient gels and then analyzed by Western blotting using anti-pRb antibody or anti-GST antibody. The bands of pRb and GSTE7 proteins were quantified by densitometry. The relative binding activities were calculated using pRb bound by wild type CPV-2 E7 GST fusion protein as 100%, and normalized with GSTE7 bands.

In order to locate potential alternative pRb binding sites in CPV-2 E7, several CPV-2 E7 truncation mutants were generated (Figure 3A). CPV-2 E7 protein was divided into CR1 (amino acids 1 to 15), CR2 (amino acids 16 to 38) and the C terminal domains (amino acids 39 to 98). The GST E7 wild type and mutant fusion proteins were purified from bacteria (Figure 3B). A binding assay with GSTE7 mutants and pRb was performed. In agreement with earlier studies [4], [14], [36], HPV16 E7CR1CR2 bound pRb much more efficiently than the HPV16 E7 carboxyl-terminus domain (Figure 3C), with 6 times more pRb being bound by the combined CR domains. In contrast, the carboxyl-terminus of CPV-2 E7 has much higher binding than the CR1 and CR2 domains, on the average 7 times higher. Importantly, similar binding affinities were also observed between the canine pRb and CPV-2 E7 (Figure 3D), indicating that these unique binding interactions were not due to differences in the species of Rb used for analysis. Thus, CPV-2 E7 uses its carboxyl-terminus instead of the CR1 and CR2 domains to associate with pRb.

### The CPV-2 E7 carboxyl-terminal domain also mediates pRb binding in vivo

**Figure 4:**
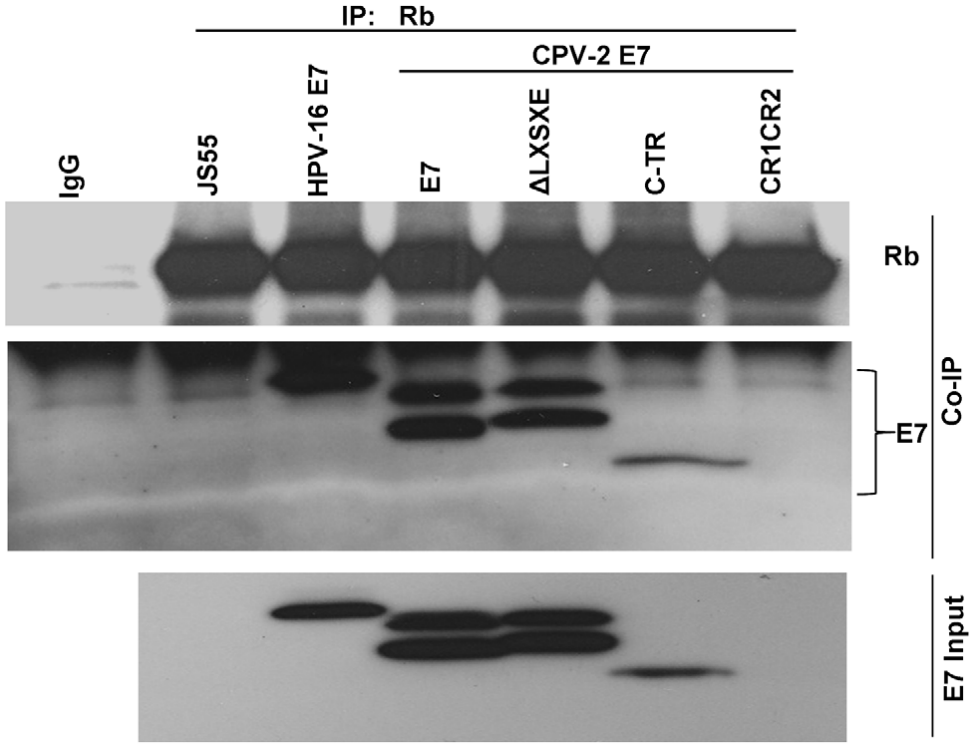
The carboxyl-terminus of canine E7 protein also mediates in vivo binding to pRb. U2OS cells were co-transfected with a series of HA-tagged E7 mutants and Rb expression plasmids. Immuno-precipitation was performed with anti-pRb antibody and the pull-down complexes were resolved on a 4 to 20% gradient gel and then analyzed by Western blotting using either anti-pRb antibody or anti-HA antibody. The total relative level of E7 proteins expressed in the transfected cells is shown in the lower panel (diluted 1∶10).

To verify that the above in vitro binding studies were relevant to in vivo conditions, U2OS cells transduced by HA-tagged CPV-2 E7 constructs were used to study the in vivo association of CPV-2 E7 protein and pRb. Co-precipitation experiments were performed with the anti-pRb antibody (Figure 4). In contrast to results with HPV 16 E7, the CPV-2 E7 mutant deleted of the LXCXE-like motif bound pRb as well as the wild-type E7 did. Furthermore, the carboxy-terminal domain of CPV-2 alone bound to pRb very well. Thus, both in vitro and in vivo studies indicate that the E7 carboxyl-terminus mediates pRb binding. The level of canine E7 CR1CR2 construct was not detectable in the cell since it appears to be unstable. The doublet band noted for both wild-type E7 and the LXCXE deletion E7 proteins is most likely due to alkylation by protease inhibitors during the IP procedure. In previous studies, we also observed two distinct forms of HPV-16 E7 and showed that they were generated in vitro by the alkylating reagents, TPCK and TLCK [37]. These reagents are used as a component of a protease-inhibitor cocktail during IP studies to prevent protein degradation. We identified cysteine 27 near the amino terminus of HPV-16 E7 as the alkylation target. In our current study, we did not observe this modification of HPV-16 E7, apparently due to interference by the amino-terminal epitope tag. In the published study where we observed alkylated HPV-16 E7, we had used an E7 protein tagged at its C-terminus. We presume that the CPV-2 E7 protein is being modified in a similar fashion, although different sites might be altered than in HPV-16 E7.

### Unlike HPV-16 E7, the CPV-2 E7 carboxyl-terminus domain is critical for destabilization of pRb

**Figure 5:**
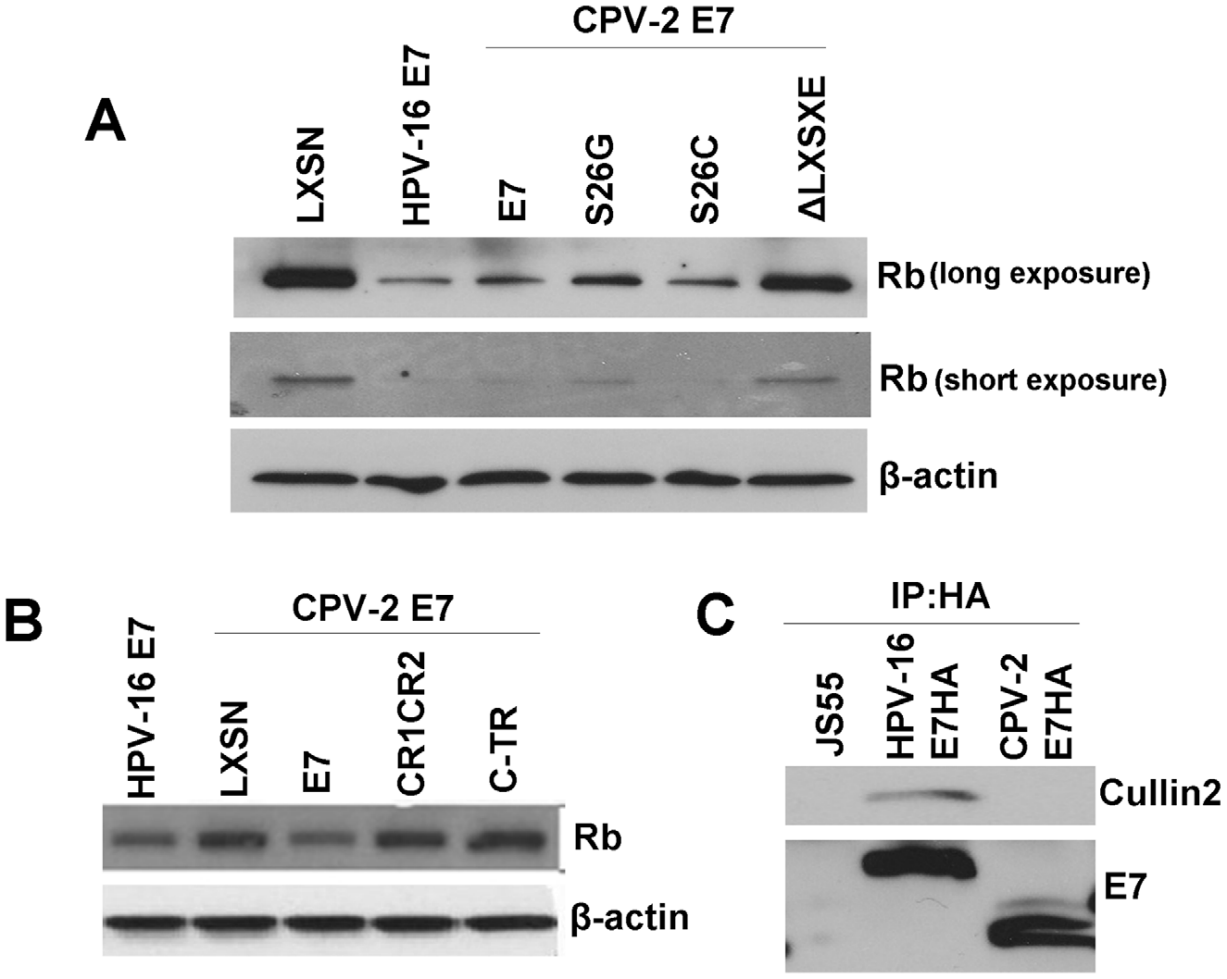
Although not the primary binding site, the canine E7 amino-terminal domain of CPV-2 E7 is important for destabilization of pRb. Keratinocytes were infected by retrovirus encoding wild type E7, E7 mutants with mutation on the LXSXE motif (A), or the domain truncation mutants (B). Cell lysates were prepared from indicated cells. Proteins were separated on a 4 to 20% gradient gel and then analyzed by Western blotting using anti-pRb antibody. (C) Cell lysates were prepared from Hela cells transfected with pJs55, pJS55-HA.HPV16 E7 or pJS55-HA.CPV-2 E7. Immuno-precipitation assay was performed with anti-HA antibody. The pulled down complexes were resolved on a 4 to 20% gradient gel and then analyzed by Western blotting using either anti-Cullin2 antibody or anti-HA antibody.

Since the mechanism used by CPV-2 E7 to bind pRb is different from that used by HPV E7, it was also possible that the two E7 proteins used alternative methods to degrade pRb. To test this possibility, a series of E7 deletion and single amino acid substitution mutants were generated. Keratinocytes were transduced with retrovirus encoding either wild type E7 or E7 mutants. Cell lysates were collected, and the level of pRb was measured with immunoblots. Surprisingly, the LXSXE-deletion mutant (which retains pRb binding) lost the ability to degrade pRb (Figure 5A). In addition, when the serine at 26 was changed to cysteine (which increases pRb binding), the degradation of pRb was not enhanced (Figure 5A). Thus, although the primary pRb binding of CPV-2 resides in the carboxyl-terminus, we observed that the amino-terminal LXSXE sequence is necessary for the degradation of pRb. Neither the E7 amino- nor carboxyl-terminal domains could independently degrade pRb (Figure 5B). This is in contrast to studies performed on high risk HPV E7 (reviewed by Munger [38]) showing that the sequences important for binding and degradation of pRb localized to CR1 and CR2. Another difference between HPV16 E7 and CPV-2 E7 is highlighted by previous studies showing that HPV16 E7 associates with the cullin 2 ubiquitin ligase complex and that this association contributes to degradation of pRb [4], [14], [36]. This does not appear to be true for CPV-2 E7 protein. Co-immunoprecipitation assays failed to detect any association of Cullin2 with CPV-2 E7 (Figure 5C).

### A gamma HPV E7 protein also binds pRb via its carboxyl-terminus

**Figure 6:**
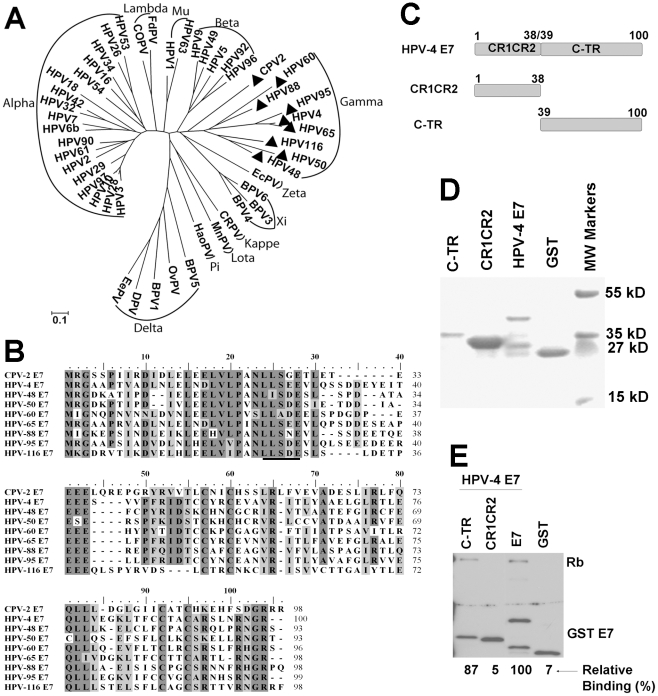
Similarities of the CPV-2 and gamma HPV E7 proteins. (A) Phylogenetic analysis of papillomavirus E7 gene sequences. The E7 gene of CPV-2 was compared with 33 HPV and 15 animal PV DNA sequences in order to establish its closest relatives. The analyzed papillomavirus genomes included were FcPV (K02019), bovine BPV1 (NC_001522), BPV2 (NC_001521), BPV3 (NC_004197)BPV4 (X05817), canine oral CPV-1 (NC_001619), Canine papillomavirus type 2 CPV-2 (AY722648), cotton rabbit CRPV (NC_001541), deer DPV (NC_001523), Equus caballus EcPV1 (AF498323), European elk EEPV (NC_001524), Felis domesticus FdPV1 (AF480454), Hamster oral HaOPV (E15110), HPV1 (NC_001356), HPV3 (NC_001588), HPV-4 (NC_001457), HPV5 (NC_001531), HPV6b (NC_000904), HPV9 (NC_001596), HPV10 (X74465), HPV16 (NC_001526), HPV18 (NC_001357), HPV26 (X74472), HPV29 (NC_001685), HPV32 (X74475), HPV34 (X74476), HPV-41 (X56147), HPV-42 (M73236), HPV-48 (NC_001690), HPV-49 (NC_001591), HPV50 (NC_001691), HPV53 (NC_001593), HPV54 (U37488), HPV60 (NC_001693), HPV61 (U31793), HPV63 (X70828), HPV90 (AY057438), HPV92 (AF531420), HPV94 (AJ620211), HPV96 (AY382779), Mastomys natalensis MnPV (NC_001605), Ovine OvPV1 (NC_001789), OvPV2 (NC_001790), Psittacus erithacus PePV (AF502599), Phocoena spinipinnis PsPV1 (NC_003348) and rabbit oral ROPV (NC_002232). (B) E7 proteins from CPV-2 and Gamma HPVs papillomaviruses were selected, and aligned using Clustal W with MEGA version 4.0. The conserved LXSXE domain is underlined. (C) Schematic representation of HPV-4 E7 proteins with mutations. HPV-4 E7 protein was divided into CR1 (amino acids 1 to 15), CR2 (amino acids 16 to 38) and C terminus domains (amino acids 39 to 100). (D) Expression and purification of bacterial expressed GST tagged E7 truncated mutants were confirmed by SDS-PAGE and Coomassie blue staining. (E) GST pull down experiments were used to analyze the binding of GST E7 constructs to pRb. Complexes were resolved on a 4 to 20% gradient gel and then analyzed by Western blotting using anti-pRb antibody or anti-GST antibody. Densitometry was used to measure the relative binding activities and normalized with GSTE7 bands.

Our current results demonstrate that at least one animal papillomavirus uses a different mechanism to bind and degrade pRb. To investigate whether some HPVs might use a similar alternative binding mechanism, we screened a papillomavirus phylogentic tree based upon the E7 protein sequence (Figure 6A). E7 proteins from 33 HPVs and 15 animal papillomaviruses were selected according to their genus [39] and aligned using Clustal W [40] with MEGA version 4.0 [32]. Based on the alignment, a phyllogenetic tree was assembled by using the minimum evolution method with MEGA version 4.0 [32]. CPV-2 E7 was most closely related to the genus gamma-papillomaviruses (HPV-4, 48, 50, 60, 65, 88, 95 and 116) and only distantly related to the genus lambda-papillomaviruses (CPV-1 and Felis domesticus papillomavirus). Interestingly, the alignment of E7 proteins revealed that all the gamma HPVs lack the LXCXE motif (Figure 6B) and, indeed, nearly all these gamma-HPVs contain the same LXSXE sequence found in CPV-2. One of the gamma-HPVs, HPV60, contains alanine rather than serine in the LXSXE sequence. To test the binding of a representative gamma-HPV E7 to pRb, HPV-4 E7 was synthesized and cloned into an expression vector. HPV-4 E7 protein was divided into CR1 (amino acids 1 to 15), CR2 (amino acids 16 to 38) and carboxyl-terminal domains (amino acids 39 to 100) (Figure 6C). Wild type HPV-4 E7 and truncation mutants were expressed as GST fusion proteins (Figure 6D) and tested for their abilities to bind pRb. Similar to CPV-2 E7, the HPV-4 E7 protein contained an LXSXE motif and bound pRb (Figure 6E). More interestingly, as shown in Figure 6E, the carboxyl- terminus of HPV-4 E7 bound pRb more efficiently than the CR1CR2 domains. It appears, therefore, that the gamma-type HPVs and CPV-2 share a mechanism by which their E7 proteins interact with pRb via the carboxyl-terminal domain.

### HPV-4 E7 is able to degrade pRb

**Figure 7:**
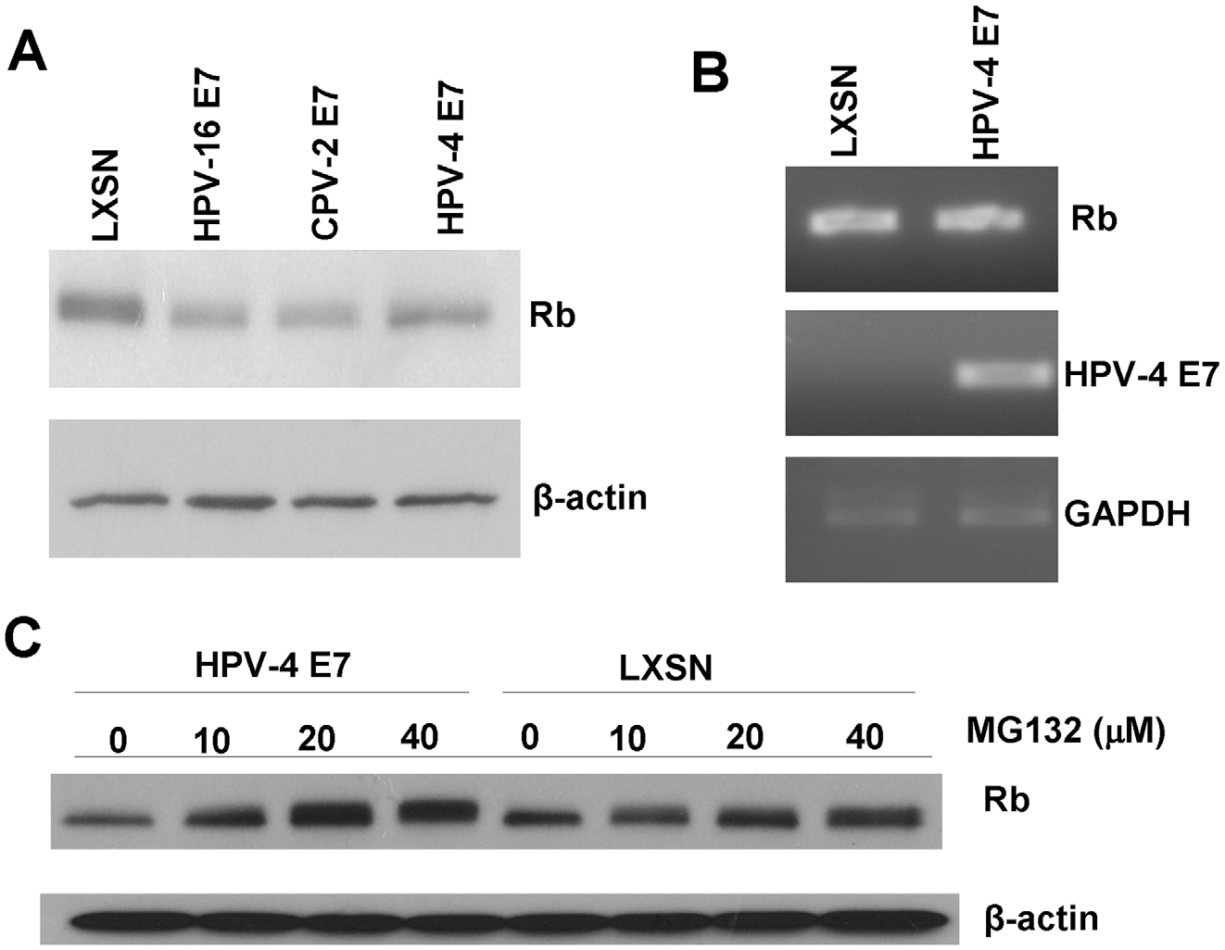
HPV-4 E7 also degrades pRb. HFKs were infected with control retrovirus or retrovirus encoding for HPV16, CPV-2 or HPV-4 E7. (A) Cell lysates were prepared from indicated cells. Proteins were separated on a 4 to 20% gradient gel and then analyzed by Western blotting using anti-pRb antibody. (B) RNA was prepared from the cells, and the levels of Rb, E7 or GAPDH mRNA were quantified by RT-PCR. (C) Cells were treated for 4 hours with proteasome inhibitor, MG132, and lysates were prepared from the indicated cells. Proteins were separated on a 4 to 20% gradient gel and then analyzed by Western blotting using anti-pRb antibody.

Due to the similar ability of the CPV-2 and HPV-4 E7 proteins to bind pRb, we also evaluated whether HPV-4 E7 could degrade pRb. In HFKs, HPV-4 E7 reduced the level of pRb in transduced cells, although somewhat less than observed with CPV-2 or HPV16 E7 (Figure 7A). To test whether the lower level of pRb protein might be due to altered gene transcription, we measured pRb mRNA levels by RT-PCR. There was no significant difference in the amount of pRb mRNA in the control cells compared to cells expressing CPV-2 E7 (Figure 7B), indicating that the pRb protein changes were post-translational. More importantly, treatment of the E7 expressing cells with proteasome inhibitor, MG132, restored the level of pRb protein (Figure 7C). These data, similar to that for CPV-2, suggest that the reduction of pRb by HPV-4 E7 is most likely the result of protein degradation.

## Discussion

Small DNA tumor viruses, such as HPV, Adenovirus, and Polyomavirus, produce viral oncoproteins that can interact with pRb and alter its function. Targeting pRb appears important for the ability of these viruses to regulate E2F and cell DNA replication and to complete the virus life cycle. All these oncoproteins, E7, E1A, and LT, use a conserved CR2 domain and LXCXE motif to bind pRb [41]. However, for CPV-2 E7, the LXCXE-like motif is not necessary for association with or degradation of pRb. It appears that CfPV has evolved an alternative mechanism to bind pRb by using the E7 carboxyl-terminal domain.

Although the HPV E7 carboxyl-terminus has been proposed to have an independent, low affinity pRb binding site [14], [36], HPV16 E7 mutants with a deletion of the LXCXE motif in CR2 fail to associate with pRb family members as determined by Western blotting and extensive proteomic analyses of associated cellular protein complexes [4]. In contrast, the carboxyl-terminus of CPV-2 E7 exhibits greater pRb binding than the CR1 and CR2 domains. Even more interesting is the finding that the CPV-2 E7 mutant deleted of the LXSXE domain still can bind to pRb with high efficiency. Furthermore, the carboxyl-terminus of CPV-2 E7 alone can bind pRb in vitro and in vivo. The carboxyl-terminus of HPV E7 has been proposed to be important for releasing E2F from pRb [20], [21]. It will be interesting to map the association site on pRb, and to determine whether the binding induces the release of E2F from pRb.

During preparation of this manuscript, three new canine papillomaviruses were identified [42]. One of the viruses, CPV7, shares high sequence homology with CPV-2 and its predicted E7 ORF encodes a protein with the LXSXE motif. Overall, however, 5 of the 7 identified canine papillomaviruses contain the LXCXE motif in their E7 protein. CPV-1 E7, which has LXCXE motif, degrades pRb in cells as anticipated (data not shown).

As shown in this study, the LXSXE motif is not limited to canine papillomaviruses; the gamma genus of HPVs also have the same motif. This genus consists of eight HPVs, 7 of which contain the E7 LXSXE motif. Interestingly, the gamma HPVs have several other similarities to CPV-2. First, they induce cutaneous rather than mucosal lesions. Second, they most likely persist in the population as subclinical infections and induce visually detectable tumors only under conditions of immunosuppression. Third, their tumor cells are characterized by histologically-distinguishable intracytoplasmic inclusion bodies [39]. The canine model may provide a new approach for studying the biology of this unique category of papillomaviruses and their stringent regulation by the host immune response.

## Supporting Information

Supporting Information S1 (0.05 MB DOC)
