# Supplementary material for: The Canine Papillomavirus and Gamma HPV E7 Proteins Use an Alternative Domain to Bind and Destabilize the Retinoblastoma Protein
Source: PLoS Pathog. 2010 Sep 2;6(9):e1001089. doi: 10.1371/journal.ppat.1001089 (PMC2932728; doi:10.1371/journal.ppat.1001089)
Supplement: Supporting Information S1 — (0.05 MB DOC) [file ppat.1001089.s001.doc]

**Supporting Information**

**Site Directed Mutagenesis**

Site directed mutagenesis was carried out using QuikChange XL Site-Directed Mutagenesis Kit (Stratagene) as specified by the manufacturer.

Prepare the sample reaction(s): 5 μl of 10× reaction buffer, 2 μl (10 ng) of dsDNA template, 1μl (125 ng) primer #1, 1 μl (125 ng) of primer #2, 1 μl of dNTP mix, 3 μl of QuikSolution, 1 μl of PfuTurbo DNA polymerase (2.5 U/μl), and ddH2O to a final volume of 50 μl. Same mixture was prepared except PfuTurbo DNA polymerase as negative control. PCR cycle each reaction using the cycling parameters outlined: 95°C 1 minute, 20 cycles (95°C 50 seconds, 60°C 50 seconds, 68°C 6 minutes), and 68°C 10 minutes. Primers of Mutagenesis were describes as table 1 below.

Table 1. List of primers for the site-directed mutagenesis

| Primers | Sequence |
| --- | --- |
| CfPVE7S26C-1 | 5'- CCTTCCAGCCAACCTGTTGTGTGGCGAAACCTTGGAGACGG -3' |
| CfPVE7S26C-2 | 5'- CCGTCTCCAAGGTTTCGCCACACAACAGGTTGGCTGGAAGG -3' |
| CfPVE7S26G-1 | 5'- CCAGCCAACCTGTTGGGCGGCGAAACCTTGGAG-3' |
| CfPVE7S26G-2 | 5'- CTCCAAGGTTTCGCCGCCCAACAGGTTGGCTGG -3' |

Add 1 μl of the *Dpn I* restriction enzyme (10 U/μl) directly to each amplification reaction (Samples and Negative control). Gently and thoroughly mix each reaction mixture by pipetting the solution up and down several times. Spin down the reaction mixtures in a microcentrifuge for 1 minute, then immediately incubate the reactions at 37°C for 1 hour to digest the parental. Then 2 μl each sample or negative control was transformed to XL10-Gold Ultracompetent Cells, 37 °C overnight. Pick up colonies to sequence to check the mutagenesis.

**Construction of E7 deletion mutants and HA-tagged E7 proteins**

Prepare the sample reaction(s): 25 μl of 2X PCR master Mix (Fementas), 1 μl (10 ng) of dsDNA template, 1μl (20μM) primer #1, 1 μl (20μM) of primer #2, and ddH2O to a final volume of 50 μl. PCR cycle each reaction using the cycling parameters outlined: 95°C 3 minute, 35 cycles (94°C 1 seconds, 52°C 45 seconds, 72°C 30 seconds), and 72°C 10 minutes. Primers of PCR were describes as table 2 below.

Table 2. List of primers for the mutagenesis on truncated mutation or HA-tagged constructs

| Primers | Sequence |
| --- | --- |
| CPV-2 E7CR1CR2-1 | 5'- GAATTCCCATGAGAGGGTCTTCGCCGATCATCAGAG -3' |
| CPV-2 E7CR1CR2-2 | 5'- GGATCCGCGGCCGCTCACTGTAACTCCTCCTCTTCCGTC -3' |
| CPV-2 E7CR3-1 | 5'- GAATTCCCATGAGAGAACCTGGGCGATACCGGGTTG -3' |
| CPV-2 E7CR3-2 | 5'- GGATCCGCGGCCGCTCACCTCCTTCGGCCATCGCTGAAG -3' |
| CPV-2 E7-1 | 5'- CGCGGATCCATGAGAGGGTCTTCGCCGATC -3' |
| CPV-2 E7-2 | 5'- CCGGAATTCTCACCTCCTTCGGCCATCGC -3' |
| HA-CPV-2 E7 (HA-CPV-2 E7CR1CR2) | 5’- ATGTACCCATACGATGTTCCAGATTACGCTAGAGGGTCTTCGCC  GATCATCAGAG -3’ |
| HA-CPV-2 E7CR3 | 5’- ATGTACCCATACGATGTTCCAGATTACGCTAGAGAACCTGGGCGA  TACCGGGTTG -3’ |
| CPV-2 E7-HA | 5’- GGATCCTCAAGCGTAGTCTGGGACGTCGTATGGGTACCTCCTTCGGCC  ATCGCTGAAG -3’ |
| HPV16 E7CR1CR2-1 | 5'- GAATTCCCATGCATGGAGATACACCTACATTGCATG -3' |
| HPV16 E7CR1CR2-2 | 5'- GGATCCGCGGCCGCTTATTCATCCTCCTCCTCTGAGC -3' |
| HPV16 E7CR3-1 | 5'- GGATCCGCGGCCGCTTATGGTTTCTGAGAACAGATG -3' |
| HPV16 E7CR3-2 | 5’- GAATTCCCATGATAGATGGTCCAGCTGGACAAGCAG -3' |
| HA-HPV16 E7 | 5’- TGTACCCATACGATGTTCCAGATTACGCTCATGGAGATACACCTACAT  TGCATG -3’ |
| HPV16 E7-HA | 5’- GGATCCTTAAGCGTAATCTGGAACATCGTATGGGTATGGTTTCTGAG  AACAGATGG -3’ |
| HPV-4 E7-1 (HPV-4 E7CR1CR2-1) | 5’- GAATTCATATGAGAGGAGCAGCGCCCACGG -3’ |
| HPV-4 E7-2  (HPV-4 E7CR3-2) | 5’- GCGGCCGCGGATCCTTATCTGCCATTTCTGTTAAGACTTC -3’ |
| HPV-4 E7CR1CR2-2 | 5’- GCGGCCGCGGATCCTTACTCATACTCATCATCTGAAG -3’ |
| HPV-4 E7CR3-1 | 5’- GAATTCATATGATTACAGAGGAGGAGTCGGTGGTTCCATTTAG -3’ |

**Reverse transcriptase PCR (RT PCR)**

RNA mixture, which was isolated from HFK cell expressing E7, was used as template to run RT PCR using ONE STEP RT-PCR KIT (QIAGEN) as specified by the manufacturer.

Thaw template RNA, primer solutions, dNTP Mix, 5x QIAGEN OneStep RT-PCR buffer, and RNase-free water, and place them on ice. Prepared a master mix for each reaction: 5x QIAGEN OneStep RT-PCR Buffer 10.0 μl, dNTP Mix (containing 10 mM of each dNTP) 2.0 μl, Primer #1 0.6 μM, Primer #2 0.6 μM, QIAGEN OneStep RT-PCR Enzyme Mix 2.0 μl, RNase inhibitor 10 units, template RNA 1.5 μg, and RNase-free water to a final volume of 50 μl. Each sample was made another copy to test DNA contamination. Program the thermal cycler according to the program: Reverse transcription: 50°C 30 minutes, then 95°C 15 minutes, 30 cycles of (94°C 30 seconds, 55 °C 30 seconds, 72°C 1 minute ), 72°C 10 minutes. Primers of RT-PCR were describes as table 3 below.

Table 3. List of RT PCR primers

| Primers | Sequence |
| --- | --- |
| Rb-RT-1 | 5'- AAATTGGATCACAGCGATACAAAC -3' |
| Rb-RT-2 | 5'- GATTCTGAGATGTACTTCTGCTATATG -3' |
| CPV-2 E7RT-1 | 5'- ATGAGAGGGTCTTCGCCGAT -3' |
| CPV-2 E7RT-2 | 5'- CGCCCAGGTTCTCTCTGTAA -3' |
| HPV-4 E7RT-1 | 5'- ATTACAGAGGAGGAGTCGGTG -3' |
| HPV-4 E7RT-2 | 5'- AAGTTGTTCCAAGGTCCGTAG -3' |
